# *GATA2* mutation in long stand *Mycobacterium kansasii* infection, myelodysplasia and MonoMAC syndrome: a case-report

**DOI:** 10.1186/s12881-019-0799-6

**Published:** 2019-04-29

**Authors:** Daniela Palheiro Mendes-de-Almeida, Francianne Gomes Andrade, Gustavo Borges, Filipe V. dos Santos-Bueno, Iracema F. Vieira, Luana Kelly M. da S. da Rocha, Daniella A. Mendes-da-Cruz, Rosely M. Zancopé-Oliveira, Rodrigo T. Calado, Maria S. Pombo-de-Oliveira

**Affiliations:** 1 Division of Hematology, Evandro Chagas National Institute of Infectology, Oswaldo Cruz Foundation, Rio de Janeiro, Brazil; 2 Pediatric Hematology-Oncology Program, 6thfloor, Research Center, Instituto Nacional de Câncer-INCa, Rua André Cavalcanti, 37, Rio de Janeiro, Zip code: 20231- 050 Brazil; 3 Department of Internal Medicine, Ribeirão Preto School of Medicine, University of São Paulo, Ribeirão Preto, Brazil; 4 Infectious Diseases Department, Hospital dos Servidores do Estado, Rio de Janeiro, Brazil; 5 Division of Hematology, Oncologia D’Or, Rio de Janeiro, Brazil; 6 Laboratory on Thymus Research, Oswaldo Cruz Institute, Oswaldo Cruz Foundation, Rio de Janeiro, Brazil; 7 Institute of Science and Technology on Neuroimmunomodulation (INCT-NIM), Oswaldo Cruz Institute, Oswaldo Cruz Foundation, Rio de Janeiro, RJ Brazil; 8 Laboratory of Mycology, Evandro Chagas National Institute of Infectology, Oswaldo Cruz Foundation, Rio de Janeiro, Brazil

**Keywords:** *GATA-2* mutation, MonoMAC syndrome, Myelodysplastic syndrome, Myelodysplasia, *Mycobacterium kansasii*

## Abstract

**Background:**

GATA2 is a transcription factor that is a critical regulator of gene expression in hematopoietic cells. GATA2 deficiency presents with multi-lineage cytopenia, mycobacterial, fungal and viral infections. Patients with *GATA2* mutation have a high risk of developing myelodysplastic syndrome or acute myeloid leukemia.

**Case presentation:**

We described a 43 years-old white male with 20-year follow-up of autoimmune and thrombotic phenomena, hypothyroidism, disseminated refractory *Mycobacterium kansasii* infection and MonoMAC syndrome. *GATA2* c.1061 C > T; p.T354 M mutation was identified after he progressed from myelodysplastic pancytopenia to refractory anemia with excess blasts type II. His relatives were also investigated and he underwent unsuccessful haematopoietic stem cell transplantation. We discuss the clinical features, genetic diagnosis and treatment of this immunodeficiency disorder.

**Conclusions:**

This case illustrates the challenge how a multidisciplinary disease should be handle. Once usual causes of immunodeficiency were excluded, clinicians should consider*GATA2* deficiency in patients with myelodysplasia and long-standing *Mycobacterium kansasii* infection.

**Electronic supplementary material:**

The online version of this article (10.1186/s12881-019-0799-6) contains supplementary material, which is available to authorized users.

## Background

Disseminated non-tuberculosis mycobacterium (NTM) infections are found in subjects with advanced human immunodeficiency virus infection, hairy cell leukaemia, or under immunosuppressive therapy regimes [1]. Host immune deficiencies with genetic disorders of the interleukin-12 (IL-12)–interferon-γ (IFN-γ) pathway has been also associated with NTM [1]. *GATA2* autosomal gene mutations are among these genetic disorders that confer predisposition NTM infections [1]. *GATA2* encodes an ill-defined protein containing two zinc-finger domains that is located in the 3p21.3 region and is a key transcriptional regulator of haematopoiesis, lymphopoiesis, and vascular development [2]. Germline loss-of-function mutations in the *GATA2* gene are associated with myelodysplastic syndrome (MDS), acute myeloid leukaemia (AML) and opportunistic infections, including NTM infections [3]. GATA2 deficiency/haploinsufficiency is an etiologic in Emberger and MonoMAC syndromes [4]. Emberger syndrome includes primary lymphedema, congenital sensorineural deafness, and cutaneous or anogenital warts [5]. MonoMAC syndrome is characterized by monocytopenia, predisposition to NTM infections, typically of the *Mycobacterium avium* complex, viral and fungal infections, pulmonary alveolar proteinosis, and natural killer (NK) and B cell deficiencies [6]. Recognition of an underlying immune defect associated with genetic susceptibility is crucial for rational treatment, preventive care, and family screening. Our aim is to describe a 20-year follow-up of a male patient finally diagnosed with MonoMAC syndrome. He had long-stand cytopenia, autoimmune and thrombotic phenomena, hypothyroidism and disseminated refractory *Mycobacterium kansasii* infection. He received anti-NTM regimen with different drugs for seven years. *GATA2* mutation was identified in the patient, and his relatives were also investigated. We discuss the clinical features, genetic diagnosis and treatment of this challenging immunodeficiency disorder.

## Case presentation

**Fig. 1:**
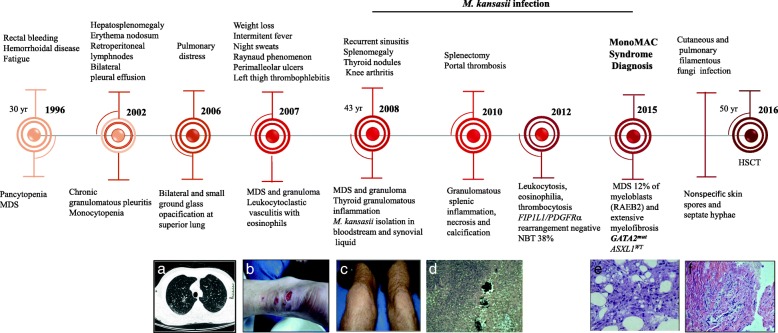
Schematic diagram summarizing the evolution of MonoMAC syndrome. Evolutional timeline of history and results of MonoMAC syndrome patient. Each ball represents a year; he initially presented pancytopenia, monocytopenia and myelodysplastic syndrome (MDS) in 1996 with 30 years-old; at 43 years-old systemic *Mycobacterium kansaii* infection developed and persisted until 2015, when refractory anemia with excess of blasts (RAEB2) and MonoMAC syndrome were diagnosed. Nitro blue tetrazolium (NBT). In 2016 he was submitted to hematopoietic stem cell transplant (HSCT) and died with 50 years-old. Pictures represents (**a**) Thoracic Computed Tomography with pulmonary opacification; **b** Painless perimalleolar ulcers; **c** Knee arthritis; **d** Granulomatous splenic inflammation, necrosis and calcification (H&E stain); **e** Bone marrow dysplasia with small and hypolobulated megakaryocytes and myelofibrosis (H&E stain of core biopsy) **f** Nonspecific skin spores and septate hyphae (Groccot strain)

**Fig. 2:**
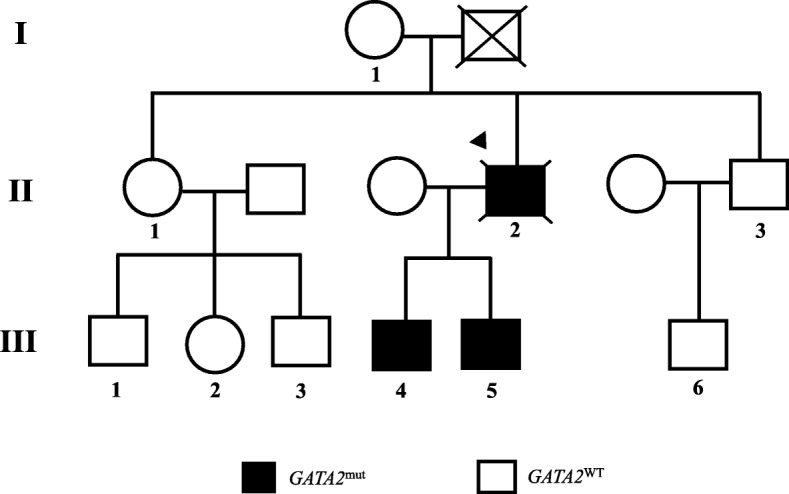
Familial pedigree Heredogram representing three generations of the family analyzed in this study. *GATA2* mutation (*GATA2*^mut^) and *GATA2* wild-type (*GATA2*^WT^) subjects are represented; the arrow indicates the proband; individuals with numbers were studied

**Fig. 3:**
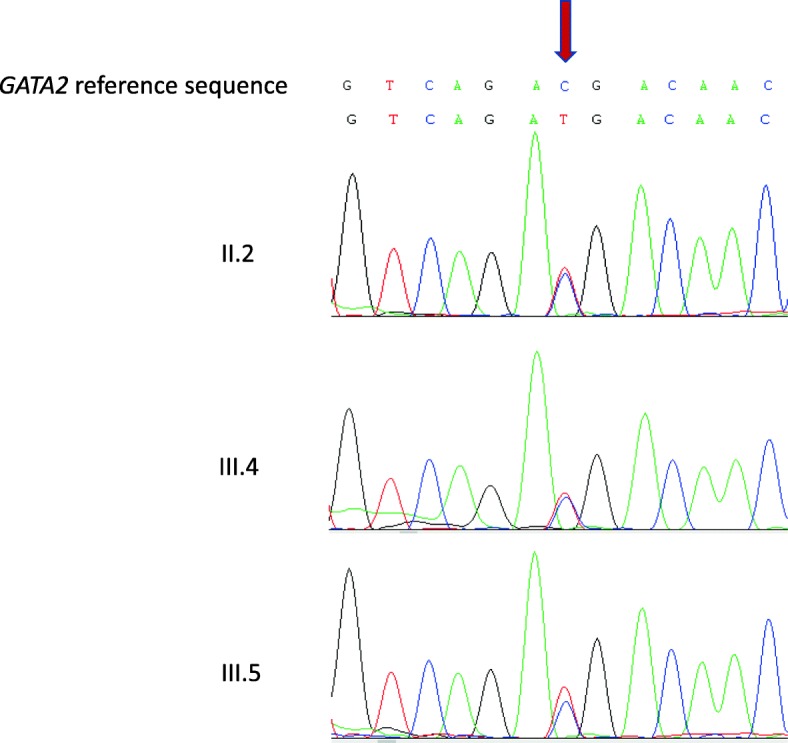
*GATA2* direct Sanger sequencing Electropherogram of *GATA2* sequencing showing the mutation c.1061 C > T; p.T354 M at the second zinc finger domain in the proband and both sons

A 43-year-old white male was seen in our clinic due to recurrent sinusitis, ankle and knee arthritis, painless nodular skin lesions at extremities, and eosinophilia in 2008. His clinical history is marked by long-standing pancytopenia and MDS diagnosed in 1996 at age 30, when he complained of spontaneous rectal bleeding and fatigue, which was diagnosed as haemorrhoidal disease (Fig. 1). Six years after the MDS diagnosis, he was admitted to the hospital with hepatosplenomegaly, erythema nodosum, retroperitoneal lymph node enlargement, and bilateral pleural effusion. Laboratory investigations failed to demonstrate any fungal, bacterial, or HIV infection. Chronic granulomatous pleuritis was discovered, and he was treated empirically for tuberculosis with standard doses of isoniazid, rifampicin, and pyrazinamide. Allergy to pyrazinamide developed, and ethambutol was used instead. Circulating blood cells demonstrated pancytopenia with low monocytes (haemoglobin, 7,7 g/dL; white blood cell (WBC) 3000 cells/μL; lymphocytes, 750/μL; monocytes, 60/μL; and platelets, 95,000/μL). One year later, monocytopenia improved slightly, but thrombocytopenia worsened (WBC, 1900 cells/μL; lymphocytes 475/μL; monocytes, 114/μL; and platelets, 33,000/μL). Seven years later (2006), developed respiratory distress and bronchial analysis was negative for bacterial infection. He was then treated with clarithromycin for possible atypical pneumonia. In 2007, a 27% decrease in total body weight loss was observed. The patient had been complaining of night fever, night sweats, Raynaud phenomenon, left thigh superficial thrombophlebitis, and painless perimalleolar ulcers. Skin and bone marrow (BM) biopsies were performed. The ulcer biopsy revealed vasculitis with eosinophils, whereas the BM biopsy showed myelodysplastic features and noncaseating granuloma, and myeloculture was negative. In 2008, he developed hypothyroidism, recurrent sinusitis, ankle and knee swellings and nodular skin lesions (Fig. 1). Antineutrophil cytoplasmic antibodies and antinuclear antibodies were within normal limits. He presented WBC 28,610/μL with marked eosinophilia (5440/μL).BM aspirate and biopsy diagnosed MDS without excess blasts. Churg-Strauss syndrome was suspected, and after 3 months of prednisone (50 mg/day), he developed arthritis and sustained night fevers. Blood culture, arthrocentesis and thyroid biopsy were performed. *Mycobacterium kansasii,* a slow-growing mycobacterium, was identified in the bloodstream and synovial fluid. The thyroid histopathological analysis demonstrated chronic and acute granulomatous inflammation. Rifampicin, isoniazid and ethambutol were restarted in addition to clarithromycin for the next 2 years. Progressive spleen enlargement culminated in splenectomy in 2010. Portal thrombosis developed at the immediate post-operatory period, and oral anticoagulant was administered. The histopathology features displayed granulomatous splenic inflammation, abscesses and central necrosis. In 2012, an increased WBC (39,080/μL) with eosinophilia (20,630/μL) and thrombocytosis (1,099,000/μL) were found. The nitro blue tetrazolium test, which is useful in diagnosing chronic granulomatous diseases, suggested a defect in phagocytosis, as it was positive in 38% of cells, and *FIP1L1/PDGFRa* rearrangement was negative, excluding hypereosinophilic syndrome. He received hydroxyurea, dexamethasone and anti-NTM therapy containing moxifloxacin until 2015, when MDS refractory anaemia with excess blasts (12%) type II (RAEB II) was diagnosed. The entire *GATA2* exons were investigated and a heterozygous germline *GATA2* (c.1061 C > T; p.T354 M) mutation was determined by Sanger sequencing of peripheral blood leukocytes (as in Additional file 1: Table S1). The combination of results led to a final diagnosis of MonoMAC syndrome. The patient was treated with 3 days of idarubicin and 7 days of cytarabine chemotherapy and developed cutaneous and pulmonary filamentous fungal infection. A skin biopsy was performed and identified nonspecific spore and septate hyphae. He was treated with liposomal B amphotericin and voriconazole, received consolidation chemotherapy with high doses of cytarabine and was submitted to haematopoietic stem cell transplant (HSCT) with a myeloablative conditioning regimen from his HLA- identical brother. He died nine months after transplantation in October 2016, in other institution, so we are not sure of the exactly cause of death. *GATA2* gene sequencing (exon 5) was performed on his relatives, including his HSCT donor and was positive only in his two healthy sons, aged 21 and 28- year-old (Figs. 2 and 3). The hotspot regions for acquired mutations exons 11–12 of *ASXL1* sequencing were also performed in the three *GATA2* mutant (GATA2^mut^) subjects but were *ASXL1* Wild-Type (*ASXL1*^WT^) as in Additional file 1: Table S1.

## Discussion and conclusions

MonoMAC syndrome was first described in 2010 in a group of 18 adult patients with disseminated NTM and other opportunistic infections [6]. Half of the patients were diagnosed with MDS/AML, characterized by reduced numbers of circulating monocytes, B cell, and NK cells [6]. Since then, patients with persistent cytopenia, even in the absence of diagnostic morphologic dysplasia, are suspected of having MonoMAC syndrome. In 2011, the MonoMAC syndrome was linked to 12 distinct *GATA2* mutations, including the T354 M in the second zinc-finger domain that was also found in our patient [5]. Although *GATA2* mutations might be found in a large number of sporadic cases, disease phenotype follows an autosomal dominant inheritance pattern [5]. To date, nearly 380 *GATA2*-deficient patients have been reported, with an estimated prevalence of myeloid neoplasia of at least 75% [7]. The most frequent clinical features described in *GATA2* deficient patients according to literature reviews and in our patient are summarized in Table 1. Non-infectious conditions also have been described in MonoMAC cases, especially endocrine, rheumatologic, and dermatological manifestations with hypothyroidism, panniculitis/erythema nodosum, arthritis and vasculitis. MonoMAC-associated MDS is usually hypocellular, shows atypical megakaryocytes, and fibrosis [8]. The 2016 revision of the World Health Organization classification for myeloid neoplasms has incorporated a subgroup of cases associated with germline mutations, that includes *GATA2* [9]. *GATA2*mut is associated with MonoMAC syndrome despite of variable GATA2 expression and initial presentation spanning from early childhood to late adulthood, with a median age of presentation of 32 years with viral, mycobacterial or fungal infections [6]. The ubiquitous influence of NTM and other infections, accompanied by co-occurrence of monocytopenia, lymphopenia, neoplasia and a vast possibility of symptoms make the initial diagnosis of MonoMAC syndrome difficult for physicians. Clinical suspicion of MonoMAC syndrome is critical to make an early genetic diagnosis and to direct an appropriate management. Other gene mutations, such as mutations in *IL12R* or *IFNγR* receptors or *STAT1*, are also important for differential diagnosis, however, CD4+, T cell and monocyte numbers are often normal in these conditions [1]. Genetic counselling should be offered to at-risk individuals [10]. The proband’s father died prematurely from a coronary artery disease (CAD). Because *GATA2* has been functionally involved in the pathophysiology of thrombosis and CAD [11], we hypothesized that *GATA2* mut would be present in the first generation of this patient’s family, but we didn’t have biological material to test him. In healthy carriers of *GATA2* mutations, antimicrobial prophylaxis with azithromycin and immunization against human papilloma virus (HPV) are suggested as follow-up treatments [10]. Other recommendations at diagnosis are screening for HPV infection and HPV-related cervical, head and neck and anogenital cancer as well as baseline pulmonary evaluation [10]. It is also important to educate patients and physicians about the increased risk of opportunistic infections, especially NTM. Vigilant BM monitoring, immunoglobulin replacement when low or with recurrent infections, screening for congenital deafness and avoidance of ototoxic drugs are all recommended [10]. Even though characterized as autosomal dominant inheritance, *GATA2* mutations are germline heterozygous and might have incomplete penetrance [12]. Allogenic HSCT has been curative for haematopoietic disease in some cases using nonmyeloablative conditioning regimens [13]. Among individuals with *GATA2* deficiency progressing to MDS/AML, acquired secondary mutations in the *ASXL1* that encoding chromatin-binding protein ASXL1 are detected in approximately 30% of cases, despite being negative in our patient (data not shown) [14]. The prognosis after MDS/AML diagnosis appears to be poor, with the best outcomes reported among individuals undergoing allogeneic HSCT in the early stages of the disease, with an overall survival rate of 57% at 36 months [4]. In conclusion, we presented a patient with long history of NTM infection, MDS, monocytopenia, autoimmune and thrombotic phenomena, hypothyroidism and carrying a *GATA* mutation. The delay in diagnosis of MonoMAC syndrome is explained by the diversity of clinical features and lack of medical knowledge by the period of disease presentation. With this report, we hope to call attention to the importance of early diagnosis of this immunodeficiency syndrome. Genetic counselling, clinical management, and HSCT in early disease stages can be safely offered.

**Table 1:** Principal clinical features of *GATA2* deficiency described previously and presented in this case report

Features^a^	Details^a^	Approximate frequency^a^	Case report presentation
MDS/AML	Early-onset, familial history, bone marrow fibrosis, aggressive disease, associated with secondary mutations	30–50% at presentation, 30 years- old median onset, 90% lifetime risk	Yes
Warts, severe Viral infection	HPV all serotypes, herpesviruses	60–70% at presentation, 10–20% disseminated CMV, VZV and EBV	No
Pulmonary alveolar proteinosis or decreased lung function	PAP (GM-CSF antibody negative), pulmonary arterial hypertension, loss of volume or diffusion, pneumonia	18% proven PAP 10% PAH 50% abnormal PFT 14% pneumonia	Yes (he had only pulmonary infiltrate)
Mycobacterial or fungal infection	NTM (MAC and others) aspergillosis, histoplasmosis	20–50% NTM 16% aspergillosis, 9% histoplasmosis	Yes
Recurrent upper respiratory tract infection	Otitis, sinusitis	10–20%	Yes
Autoimmune manifestations	Panniculitis, arthritis, lupus-like, hypothyroidism, hepatitis/PBC	30% panniculitis, arthritis in up to 50% overall	Yes
Solid malignancy	HPV and EBV- mesenchymal related, breast, prostate and kidney cancer, metastatic melanoma	20–35% intra-epithelial neoplasia, 22% of women with > 35 years breast cancer, other skin cancer 10%	No
Lymphedema	Childhood or adolescence	11–20%	No
Thrombosis	DVT, PE, Catheter-related	25% risk overall	Yes
Deafness	Neurosensorial	20% abnormal audiograms	No

*MDS* myelodysplastic syndrome, *AML* acute myeloid leukaemia, *HPV* human papilloma virus, *CMV* cytomegalovirus, *EBV* Epstein– Barr virus, *VZV* varicella zoster virus, *PAP* pulmonary alveolar proteinosis, *GM-CSF* granulocyte-macrophage colony-stimulating factor, *PAH* pulmonary arterial hypertension, *PFT* pulmonary function tests, *NTM* nontuberculous mycobacteria, *MAC Mycobacterium avium* complex, *PBC* primary biliary cirrhosis, *DVT* deep vein thrombosis, *PE* pulmonary embolism. ^a^According to Collin et al., 2015

## Additional file

Additional file 1: **Table S1.** *GATA2* and *ASXL1* genes conditions, oligoprimers and sequences and PCR conditions for Sanger Sequencing. (DOCX 18 kb)

## Abbreviations

AML: Acute myeloid leukaemia
BM: Bone marrow
CAD: Coronary artery disease
HPV: Human papilloma virus
HSCT: Haematopoietic stem cell transplant
IFN- γ: Interferon-γ
IL-12: Interleukin-12
MDS: Myelodysplastic syndrome
NK: Natural killer
NTM: Disseminated non-tuberculosis mycobacterium
RAEB2: Anaemia with excess blasts type 2
WBC: White blood cell
