# Supplementary material for: GATA2 mutation in long stand Mycobacterium kansasii infection, myelodysplasia and MonoMAC syndrome: a case-report
Source: BMC Med Genet. 2019 Apr 29;20:64. doi: 10.1186/s12881-019-0799-6 (PMC6489290; doi:10.1186/s12881-019-0799-6)
Supplement: Supplementary file 1 — Table S1. GATA2 and ASXL1 genes conditions, oligoprimers and sequences and PCR conditions for Sanger Sequencing. (DOCX 18 kb) [file 12881_2019_799_MOESM1_ESM.docx]

| Gene amplicon/ Size PCR product (bp) | Primers | Sequence oligonucleotide  foward and reverse 5`-3` | PCR conditions |
| --- | --- | --- | --- |
| *GATA2* exon 1  378 bp | *GATA2* ex1 F | GTGAGCGCCAGGAAGGTA | 94°C for 1 min, 35 cycles of denaturation at 94°C for 30sec, annealing at 62º. C for 30 sec, and 64°C for 30sec; and a final extension at 72°C for 1 min |
|  | *GATA2* ex1R | AAACGGACCAAGCGATTC |  |
| *GATA2* exon 2  400 bp | *GATA2* ex2 F | ACCTCGTGGTGGGACTTTG | 94°C for 4 min, 35 cycles of denaturation at 94°C for 20 sec, annealing at 60º. C for 30 sec, and 72°C for 1 min; and a final extension at 72°C for 3 min |
|  | *GATA2* ex2 R | GATCCTACATCCGGGGAAGC |  |
| *GATA2* exon 3A  412 bp | *GATA2* ex3A F | GTCCCTAGCTCTGCCTACCC | 94°C for 4 min, 35 cycles of denaturation at 94°C for 20 sec, annealing at 60º. C for 30 sec, and 72°C for 1 min; and a final extension at 72°C for 3 min |
|  | *GATA2* ex3B R | CTCCTCGGGCTGCACTAC |  |
| *GATA2* exon 3B  406 bp | *GATA2* ex3B F | ACCTTTTCGGCTTCCCAC | 94°C for 4 min, 35 cycles of denaturation at 94°C for 20 sec, annealing at 60º. C for 30 sec, and 72°C for 1 min; and a final extension at 72°C for 3 min |
|  | *GATA2* ex3B R | CTCTCCCAAGTCACAGCTCC |  |
| *GATA2* exon 4  229 pb | *GATA2* ex4 F | GACTCCCTCCCGAGAACTTG | 94°C for 4 min, 35 cycles of denaturation at 94°C for 20 sec, annealing at 58º. C for 30 sec, and 72°C for 1 min; and a final extension at 72°C for 3 min |
|  | *GATA2* ex4 R | TGTAATTAACCGCCAGCTCC |  |
| *GATA2*exon 5  223 bp | *GATA2* ex5F | GTGGAGCGAGGGTCAGG | 94°C for 1 min, 35 cycles of denaturation at 94°C for 30 sec, annealing at 64°C for 30sec, and 72°C for 1 min; and a final extension at 72°C for 3 min |
|  | *GATA2* ex5R | CACAAAGCGCAGAGGTCC |  |
| *GATA2* exon 6  415 bp | *GATA2* ex6 F | AGGAATGTTGCTGGAGGAAG | 94°C for 2 min, 35 cycles of denaturation at 95°C for 30 sec, annealing at 60º. C for 30 sec, and 72°C for 45 sec; and a final extension at 72°C for 3 min |
|  | *GATA2* ex6 R | GCTGGCAGGAGTGGTGTC |  |
| *ASXL1*exon 11  724 bp | *ASXL1* ex11F | GGCCTGAAACTGATGGCTGTGATT | 95 °C for 10 min; 35 cycles of denaturation at 95°C for 30 s, annealing at 55°C for 30 sec, and 72°C for 30 sec; and a final extension at 72° C for 10 min |
|  | *ASXL1* ex11R | TGACCCTCAAAGAAAACCTGGCTC |  |
| *ASXL1*exon 12 PCR1  561 bp | *ASXL1* ex12F | AGGTCAGATCACCCAGTCAGTT | 95 °C for 10 min; 35 cycles of denaturation at 95°C for 30 s, annealing at 55°C for 30 sec, and 72°C for 30 sec; and a final extension at 72° C for 10 min |
|  | *ASXL1* ex12R | TAGCCCATCTGTGAGTCCAACTGT |  |
| *ASXL1*exon 12 PCR2  558 bp | *ASXL1* ex12F | AGAGGACCTGCCTTCTCTGAGAAA | 95 °C for 10 min; 35 cycles of denaturation at 95°C for 30 s, annealing at 55°C for 30 sec, and 72°C for 30 sec; and a final extension at 72° C for 10 min |
|  | *ASXL1* ex12R | TTCGATGGGATGGGTATCCAATGC |  |
| *ASXL1*exon 12 PCR3  532 bp | *ASXL1* ex12F | ACTTGAAAACCAAGGCTCTCGT | 95 °C for 10 min; 35 cycles of denaturation at 95°C for 30 s, annealing at 55°C for 30 sec, and 72°C for 30 sec; and a final extension at 72° C for 10 min |
|  | *ASXL1* ex12R | GCAACCATCCCATCTGTCCTTGTA |  |
| *ASXL1*exon 12 PCR4  674 bp | *ASXL1* ex12F | GGTGGACAAGGATGAGAAACCCAA | 95 °C for 10 min; 35 cycles of denaturation at 95°C for 30 s, annealing at 55°C for 30 sec, and 72°C for 30 sec; and a final extension at 72° C for 10 min |
|  | *ASXL1* ex12R | TGTCCTGTGACATAGCACGGACTT |  |
| *ASXL1*exon 12 PCR5  533 bp | *ASXL1* ex12F | TGGATTCCAAAGAGCAGTTCTCTTC | 95 °C for 10 min; 35 cycles of denaturation at 95°C for 30 s, annealing at 55°C for 30 sec, and 72°C for 30 sec; and a final extension at 72° C for 10 min |
|  | *ASXL1* ex12R | CATGACAAAGGGCATCCCTTCCAA |  |
| *ASXL1*exon 12 PCR6  593 bp | *ASXL1* ex12F | ACAGGAAAGCTACTGGGCATAGTC | 95 °C for 10 min; 35 cycles of denaturation at 95°C for 30 s, annealing at 55°C for 30 sec, and 72°C for 30 sec; and a final extension at 72° C for 10 min |
|  | *ASXL1* ex12R | CAAGAGTGCTCCTGCCTAAAGAGT |  |

For *GATA2* PCR and sequencing: Genomic DNA was purified from blood and bone marrrow samples with QIAamp® DNA Blood Mini Kit (Qiagen, Hilden, Germany). Mutations were determined in the entire coding region on the *GATA2,* according to Wang et al, 2015, after amplification of 100 ng of genomic DNA by polymerase chain reaction (PCR) with 1U of Taq DNA polimerase (Invitrogen, Carlsbad, CA), 1x PCR buffer (50 mM KCl, 20nM Tris–HCl pH 8,4); 1,5 mM of MgCl_2_; 0,2 mM of dNTPs, and 0.4 μM each forward and reverse primer. The fragments were visualized on a 1.5% agarose gel stained with GelRed® (Biotium, CA, USA). Fragments were purified GFX® PCR DNA and Gel Band Purification Kit (GE Healthcare, NE, UK) according to manufacturer’s instructions. DNA sequencing was performed with BigDye® Terminator v3.1 Cycle Sequencing Kit, Applied Biosystems, CA, USA) in the ABI3130xl Genetic Analyzer (Applied Biosystems, CA, USA). Sequences were compared with reference sequence (GATA2 NG 9334.1.gbk).For *ASXL1* PCR and sequencieng: PCR amplifications were done in a total volume of 50 µl PCR mix containing at least 100 ng template DNA, buffer 1x (50mM KCl; 20nM Tris-HCl, pH 8,4) (Invitrogen, CA, USA), 1,5mM of MgCl_2_, 200 µmol of each deoxynucleotide triphosphate, 20 pmol of each primer and 1 unitTaq DNA polimerase (Invitrogem, CA, USA). The primers are described in Supplemental Table 1. After the PCR reaction, the products were visualized on 2% agarose gels by electrophoresis.
